# Small Molecules Detected by Second-Harmonic Generation Modulate the Conformation of Monomeric α-Synuclein and Reduce Its Aggregation in Cells*

**DOI:** 10.1074/jbc.M114.636027

**Published:** 2015-09-22

**Authors:** Ben Moree, Guowei Yin, Diana F. Lázaro, Francesca Munari, Timo Strohäker, Karin Giller, Stefan Becker, Tiago F. Outeiro, Markus Zweckstetter, Joshua Salafsky

**Affiliations:** From ‡ Biodesy, Inc., South San Francisco, California 94080,; the § Max Planck Institute for Biophysical Chemistry, Am Fassberg 11, 37077 Göttingen, Germany,; the ¶ Center for Nanoscale Microscopy and Molecular Physiology of the Brain, University Medical Center, 37075 Göttingen, Germany,; the ‖ Department of Neurodegeneration and Restorative Research, University Medical Center Göttingen, 37073 Göttingen, Germany, and; the ** German Center for Neurodegenerative Diseases (DZNE), 37077 Göttingen, Germany

**Keywords:** drug discovery, Parkinson disease, protein aggregation, protein conformation, protein drug interaction

## Abstract

Proteins are structurally dynamic molecules that perform specialized functions through unique conformational changes accessible in physiological environments. An ability to specifically and selectively control protein function via conformational modulation is an important goal for development of novel therapeutics and studies of protein mechanism in biological networks and disease. Here we applied a second-harmonic generation-based technique for studying protein conformation in solution and in real time to the intrinsically disordered, Parkinson disease related protein α-synuclein. From a fragment library, we identified small molecule modulators that bind to monomeric α-synuclein *in vitro* and significantly reduce α-synuclein aggregation in a neuronal cell culture model. Our results indicate that the conformation of α-synuclein is linked to the aggregation of protein in cells. They also provide support for a therapeutic strategy of targeting specific conformations of the protein to suppress or control its aggregation.

## Teaser

**Background:** α-Synuclein aggregation is associated with Parkinson disease.

**Results:** Small molecules were identified by second-harmonic generation (SHG) that change α-synuclein conformation *in vitro* and reduce the aggregation of protein in cells.

**Conclusion:** Conformation plays a role in α-synuclein aggregation in cells.

**Significance:** Small molecules that modulate the conformation of α-synuclein and reduce its aggregation could be developed into therapeutics for Parkinson disease.

## Introduction

The regulation of protein activity and function through changes in protein conformation is a central theme in biology. Diverse biological processes such as enzyme catalysis, allostery, and protein-protein interactions are all governed by conformational changes (1–3). Classical biochemical experiments on enzyme catalysis led to the development of the structure-function paradigm, whereby the structure of a protein determines its function (4, 5). However, this paradigm has been challenged by the discovery of intrinsically disordered proteins (IDPs)^4^ (6, 7).

IDPs exist as a highly dynamic ensemble of conformations and thus do not populate a dominant and stable three-dimensional structure. IDPs as a class are estimated to comprise ∼15–45% of all eukaryotic proteins and include both proteins that lack a folded structure as well as those that have disordered regions, such as loops and linkers, of greater than 30 amino acids in length (8). Although they are natively unstructured, IDPs may adopt secondary and tertiary structures upon binding ligands or other proteins (9).

The protein α-synuclein is a 140-residue IDP that is highly enriched in the brain (10). Although the precise cellular function of α-synuclein is not completely understood, it is thought to promote SNARE complex assembly and play a role in regulating synaptic vesicle levels and dopamine release at the presynaptic terminal (11–13). The aggregation and accumulation of α-synuclein in neurons is associated with a number of neurodegenerative diseases known as synucleinopathies. One such example is Parkinson disease (PD), which is a characterized by the progressive loss of dopaminergic neurons in the *substantia nigra* of the midbrain and by the presence of Lewy bodies, which are comprised mainly of aggregated α-synuclein fibrils (14). Mutations in α-synuclein have also been linked to both sporadic and rare forms of early onset PD, providing further evidence that the protein is implicated in the pathogenesis of the disease (15–18). *In vitro*, α-synuclein adopts a wide range of conformations from compact to fully extended (19–21). Interactions between the N and C termini appear to stabilize the protein predominantly in a compact, monomeric conformation that is non-toxic (19). The process by which the protein proceeds from monomer to oligomers and fibrillar aggregates is currently the subject of intense scrutiny (22–24). Growing evidence suggests that oligomers of α-synuclein, formed prior to the larger aggregates found in the brains of patients with the disease, may be neurotoxic *in vivo* (20, 22). Irrespective of the toxic species, monomeric α-synuclein is an attractive therapeutic target for small molecules that modulate α-synuclein conformation as it is the most upstream form of the protein in the aggregation process. However, the intrinsic disorder of the protein makes it difficult to screen by conventional techniques and few molecules are known to bind specifically to the protein (25–32).

**FIGURE 1.**
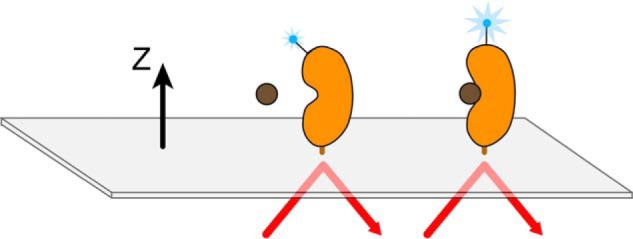
**Schematic of second-harmonic generation.** Incident laser light strikes the surface and through total internal reflection creates an evanescent wave that decays away from the surface. Labeled protein is bound to the surface and the SHG signal magnitude depends on the orientation of the dye label relative to the surface normal (*z* axis). A conformational change that re-orients the average, net orientation of the label toward or away from the normal results in a signal change.

To overcome the challenges associated with traditional small molecule screening of α-synuclein, we developed a novel second-harmonic generation (SHG)-based screen to identify compounds that directly modulate the conformation of monomeric α-synuclein. SHG is a nonlinear optical technique (33, 34) in which two photons of equal energy are combined by a nonlinear material or molecule to generate one photon with twice the energy. Because the intensity of the SHG signal is highly sensitive to the angular orientation of second-harmonic active molecules tethered to a surface, the technique can be applied to study structure and conformational changes. (35–38). Although biological molecules are not usually second-harmonic active, they can be rendered so through the incorporation of a second-harmonic active dye molecule (39). Once tethered to a surface, a labeled second-harmonic active protein irradiated by a fundamental light beam produces an SHG signal whose intensity depends sensitively on the tilt angle of the dye with respect to the surface (Fig. 1). The SHG intensity is the coherent superposition of scattered second-harmonic light orientationally averaged across the molecules in the irradiated ensemble. When the protein molecules undergo a conformational change upon ligand binding, this causes a change in the orientational distribution of the second-harmonic active moiety, leading to a change in the intensity of light. Changes in SHG intensity therefore correspond with high orientational sensitivity to conformational changes at the attachment site of the second-harmonic active probe. In addition, conformational changes can be classified by their response in both magnitude and direction relative to baseline (for a more detailed explanation of the theoretical background for SHG, please see the “Experimental Procedures”).

In the study presented here, we used an SHG-based fragment screen to identify novel conformational modulators of α-synuclein. We subsequently used these modulators, which include a compound we named BIOD303 and its analogs, to probe the biological chemistry of monomeric α-synuclein. We demonstrate that these molecules bind to the protein by both SHG and NMR spectroscopy and by paramagnetic relaxation enhancement (PRE). We also show definitively by SHG that these molecules induce a conformational change in α-synuclein. Finally, we demonstrate that these modulators reduce α-synuclein aggregation in a human neuronal cell model. Taken together, our results demonstrate that modulation of α-synuclein conformation by small molecules can significantly reduce the aggregation of the protein in cells. In addition, our results demonstrate that SHG can sensitively study the biological mechanism of ligand-protein interactions and enable the identification of novel ligands for drug discovery and basic research.

## Experimental Procedures

### 

#### 

##### α-Synuclein Purification and Labeling

A single-site cysteine mutant (A90C) of α-synuclein was constructed and labeled with the second-harmonic active dye SHG2-maleimide (thiol-reactive, available from Biodesy), and conjugated to the protein according to the manufacturer's instructions. The incorporation of a single label at A90C was confirmed by mass spectrometry.

##### SHG Instrumentation and Measurements

Our instrument comprises a mode-locked Ti:Sapphire oscillator, which provides the fundamental beam necessary to generate the second-harmonic signal (high peak power). For these experiments, we used a Mira 900 Ti:Sapphire ultrafast oscillator (Coherent Inc., Santa Clara, CA) pumped by a Millenia V DPSS laser (Spectra-Physics Corp., Santa Clara, CA). The fundamental was passed through a half-wave plate to select *p-*polarization (used for all the experiments described here), and focused into a Dove prism for total internal reflection at a spot size of ∼100 μm. The second-harmonic light was collected by a lens, separated from the fundamental using a dichroic mirror and wavelength filters, and directed into a PMT module with a built-in pre-amplifier for photon counting (Hamamatsu, Bridgewater, NJ). A custom electronics board was used to digitize the signal and the data were sent to a computer running customized control and data collection software (Labview, National Instruments Corp., Austin, TX). For these experiments, a microscope slide with protein was coupled to a prism using BK7 index matching fluid (Cargille, Cedar Grove, NJ) and the prism itself was secured onto a one-dimensional translation stage capable of 1-μm randomly addressable precision (Renishaw, Parker-Hannifin Corp., Rohnert Park, CA). A silicone template defined 16 wells on each slide. Each of the 16 wells is read in sequence by translating the stage via the control software. The instrument was also outfitted with a single-channel liquid injector (Cavro, Tecan Systems, Inc., San Jose, CA) that delivered a liquid bolus (in the screening experiments, spermine).

##### α-Synuclein Assay Slide Preparation

A custom 2-mm thick silicone template with adhesive backing (Arrowleaf Research, Bend, OR) was applied to commercially available aldehyde derivatized glass microscope slides (Xenobind, Xenopore, Hawthorne, NJ). Each silicone template defined 16 wells whose spacing and diameter equals those of a standard 384-well plate format. Stock solutions of second-harmonic active dye-labeled α-synuclein were diluted to 5 μm final concentration (below the threshold for oligomer formation (40)) in PBS and allowed to adsorb to the glass surface in each well by incubating at room temperature for 30 min. After allowing the protein to adsorb, the slide was placed on the instrument and each well was scanned via SHG to determine the amount of signal in each well. Unbound protein was then removed by washing five times with 20 μl of PBS. A post-wash well scan was then performed.

##### Maybridge Library Screening

The Maybridge Rule of Three (Ro3) fragment library contains 1,000 pharmacophores of ≥95% purity that conform to the rule of three standard (molecular mass <300 Da, ClogP <3, and the number of hydrogen bond donor and acceptors <3) (41). Each compound has a stated solubility of up to 1 mm in PBS. Because fragments typically possess lower potency than larger compounds, they were screened at 1 mm final concentration. For screening experiments, each compound from the library was solubilized at 50 mm in DMSO and diluted in PBS to produce a final concentration of 2 mm (4% DMSO). Each compound (2 mm) was then injected into a well containing an equal volume of buffer to produce a final concentration of 1 mm compound in the well. To ensure that a buffer mismatch did not occur, the buffer used during screening was PBS + 4% DMSO. The slide was then loaded onto the instrument stage, baseline signal was measured in real time, and spermine was injected at 5 mm while reading the SHG signal. In these experiments, baseline signals were monitored for 8 s, followed by spermine injection, and the SHG signal was recorded for an additional 8 s. For the screen, each well was loaded with 5 μm α-synuclein, washed out after binding to the surface, and preincubated with one compound from the Maybridge Ro3 library for 30 min at 1 mm. Spermine was then added to each well at 5 mm and the resulting change in SHG signal was measured. Compounds that inhibited the spermine-induced conformational change as measured by SHG were re-tested. A similar procedure was used to test the BIOD303 analogs.

##### Dose-response Curves

To measure the dose-response curvesof spermine (Sigma) and BIOD303, serial dilutions of each concentrated stock compound were prepared. Each compound was added to a well to produce the indicated final concentration. For spermine, the range of concentrations tested was 10 μm, 30 μm, 100 μm, 300 μm, 1 mm, 3 mm, and 10 mm. For BIOD303 the concentration range was 5 μm, 32.5 μm, 62.5 μm, 125 μm, 250 μm, 500 μm, and 1 mm. Concentrations were then converted to logarithmic scale and the percent change at each concentration was plotted in Prism (GraphPad Software, La Jolla, CA) using a non-linear regression fit for log (agonist) *versus* normalized response. The data were normalized so that the values for the lowest concentration and the highest concentration were set to 0 and 100%, respectively. EC_50_ values were calculated empirically using the software.

##### Compound Injections

BIOD303, spermine, spermidine, and phthalocyanine tetrasulfonate (PcTS, Sigma) were all freshly prepared as stock solutions at the indicated concentrations in PBS. Compounds from the Maybridge library were also prepared in PBS at the indicated concentrations. To determine the change in SHG signal that a given compound produced, the baseline signal of α-synuclein was measured for 10 s followed by compound injection. The change in SHG intensity was recorded for 90 s after compound addition. Where appropriate, the buffer was supplemented with DMSO to prevent buffer mismatch.

##### Quantifying Changes in SHG Intensity

Control experiments were performed on each compound to empirically determine the time required for each compound to produce a maximal response (*t*_max_). For all experiments except those using PcTS the maximal response measured by SHG occurred within 30 s after compound addition. For the PcTS experiments, the maximal response occurred ∼45 s after compound addition. To calculate the percent change in SHG intensity (%Δ_SH_), the second-harmonic intensity measured just prior to injection (*I_t_*_0_) was subtracted from the second-harmonic intensity at *t*_max_ (*I_t_*_max_) and then divided by the initial second-harmonic intensity (*I_t_*_0_) according to Equation 1.

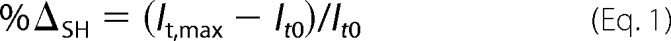


All experiments included a control buffer injection that was used to determine the threshold for SHG intensity change, which was calculated in a similar manner. The net SHG intensity change for each compound was then reported by subtracting the buffer threshold value from the SHG intensity value produced by compound addition. The resulting value was reported as the percent change in SHG intensity resulting from compound addition.

##### NMR Spectroscopy

NMR spectra were acquired at 15 °C on a Bruker Avance 600 NMR spectrometer using a triple-resonance cryoprobe equipped with *z* axis self-shielded gradient coils. α-Synuclein and BIOD303 or the analog compounds were dissolved in 50 mm HEPES, 100 mm NaCl buffer containing 2% DMSO. Low temperature (15 °C) inhibits the rate of aggregation of α-synuclein and ensures the species under study is in the monomeric form. α-Synuclein-ligand binding was measured using two-dimensional ^1^H-^15^N heteronuclear single quantum coherence experiments. Chemical shift perturbations Δσ^1^H^15^N were calculated according to [(Δσ^1^H)^2^ + (0.2Δσ^15^N)^2^]^0.5^, where Δσ^1^H and Δσ^15^N are the observed changes in the ^1^H and ^15^N dimensions. PRE was measured using spin-labeled A90C α-synuclein. Spin labeling with oxy-2,2,5,5-tetramethyl-d-pyrroline-3-methyl)-methanethiosulfonate (MTSL, Toronto Research Chemicals, Toronto) was carried out as described (42). PRE effects were measured from the peak intensity ratios between two ^1^H-^15^N heteronuclear single quantum coherence spectra in the absence and presence of a 10-fold excess (compared with protein) of DTT. To exclude contributions from DMSO, the chemical shifts and PREs in the presence of ligand were compared with those observed upon addition of the same amount of DMSO without ligand. Data processing was performed using the software packages NMRPipe/NMRDraw (43), Topspin (Bruker), and Sparky (62).

##### Cell Culture

Human neuroglioma cells (H4) were maintained in Opti-MEM I Reduced Serum Medium (Life Technologies-Gibco) supplemented with 10% fetal bovine serum Gold (FBS) (PAA, Cölbe, Germany) at 37 °C in an atmosphere of 5% CO_2_.

##### Cell Transfection

H4 Cells were plated in 12-well plates (Costar, Corning, New York) 1 day prior to transfection. On the subsequent day, cells were transfected with FuGENE® 6 Transfection Reagent (Promega, Madison, WI) according to the manufacturer's instructions with equal amounts of plasmids encoding a C terminally modified α-synuclein (SynT construct) and synphilin-1 as previously described (44–46). Twenty-four hours after the transfections, the cells were treated with different compounds at different concentrations (0, 10, 100, or 500 μm). DMSO was used as the vehicle. Twenty-four hours later the cells were subjected to immunocytochemistry to examine α-synuclein inclusion formation.

##### Immunocytochemistry

After transfection, cells were washedwith PBS and fixed with 4% paraformaldehyde for 10 min at room temperature. Cells were then permeabilized with 0.5% Triton X-100 (Sigma) for 20 min at room temperature and blocked in 1.5% normal goat serum (PAA)/PBS for 1 h. Cells were then incubated for 3 h with mouse anti-ASYN primary antibody (1:1000, BD Transduction Laboratories, NJ), and afterward with a secondary antibody (Alexa Fluor 488 donkey anti-mouse IgG) for 2 h at room temperature. Finally, cells were stained with Hoechst 33258 (Life Technologies-Invitrogen) (1:5000 in PBS) for 5 min and maintained in PBS for epifluorescence microscopy.

##### Quantification of α-Synuclein Inclusions

Experiments were performed as previously described (44). Briefly, transfected cells were detected and scored based on the α-synuclein inclusions pattern and classified as presented. Results were expressed as the percentage of the total number of transfected cells. A minimum of 50 cells were counted per condition.

##### Immunoblotting

Twenty-four hours after H4 cells co-expressing α-synuclein (SynT) and synphilin-1 were treated with BIOD303, the cells were lysed with radioimmunoprecipitation assay (RIPA) lysis buffer (50 mm Tris, pH 8.0, 0.15 m NaCl, 0.1% SDS, 1% Nonidet P-40, 0.5% sodium deoxycholate), 2 mm EDTA and a protease inhibitor mixture (1 tablet/10 ml, Roche Diagnostics, Mannheim, Germany). Using the Bradford assay (Bio-Rad Laboratories), the protein concentration was determined and the gels were loaded with 30 μg of protein. The samples were denatured for 5 min at 99 °C in protein sample buffer (125 mm Tris-HCl, pH 6.8, 4% SDS 0.5% bromphenol blue, 4 mm EDTA, 20% glycerol, 10% β-mercaptoethanol).

The samples were separated on 12% SDS-PAGE with a constant voltage of 110 V using Tris glycine, SDS 0.5% running buffer (250 mm Tris, 200 mm glycine, 1% SDS, pH 8.3) for 90 min and transferred to a nitrocellulose membrane (Protran, Schleicher and Schuell, Whatman GmbH, Dassel, Germany) for 120 min with constant current at 0.3 A using Tris glycine transfer buffer.

Membranes were blocked with 5% (w/v) skim milk (Fluka, Sigma) in 1× TBS-Tween (50 mm Tris, 150 mm NaCl, 0.05% Tween, pH 7.5) for 60 min at room temperature. Afterward, membranes were incubated with the primary antibody, mouse anti-ASYN (1:1000, BD Biosciences, San Jose, CA), and 1:5000 mouse anti-GAPDH (Cell Signaling, Danvers, MA) in 3% albumin bovine fraction V/TBS (NZYTech, Lisbon, Portugal) overnight at 4 °C. After washing three times in TBS-Tween, the membranes were incubated for 2 h with secondary antibody, either anti-mouse IgG- or anti-rabbit IgG-horseradish peroxidase (GE Healthcare, Bucks, United Kingdom) at 1:10,000 in 3% milk/TBS-Tween. Detection was performed using Luminol Reagent and peroxide solution (Millipore, Billerica, MA). Protein levels were quantified using ImageJ and normalized to the GAPDH levels.

##### Proteasome Activity Reporter Assay

H4 cells expressing SynT + synphilin were transfected with a plasmid encoding GFP-u, a reporter of proteasome activity (47). After 24 h, cells were treated with different concentrations of BIOD303. Cells were imaged using an Olympus IX81-ZDC microscope system (Olympus Germany, Hamburg, Germany) and analyzed using the Olympus Scan∧R Image Analysis Software.

##### Triton X-100 Solubility Assay

Experiments were performed as previously described (44). Briefly, H4 cells transiently co-transfected with α-synuclein (SynT) and synphilin-1 were treated with vehicle (DMSO) or BIOD303 (10, 100, or 500 μm). After 24 h, cells were washed with cold PBS and lysed in lysis buffer (PBS supplemented with protease and phosphatase inhibitor mixture tablets, Roche Applied Science). Samples were sonicated three times for 30 s and incubated on ice for 1 min between each sonication step. Total protein concentration was measured and 1% of Triton X-100 was added to 150 μg of the protein extracts. Samples were incubated at 4 °C for 30 min and the Triton X-100-insoluble (InsoI) fraction was separated from the soluble (Sol) fraction by centrifugation at 100,000 × *g* for 1 h at 4 °C. The insoluble fraction was resuspended in 40 μl of lysis buffer containing 2% SDS and sonicated twice for 30 s with 1-min incubation on ice between each sonication step. Both fractions were run on an SDS-PAGE for immunoblotting analysis. A representative membrane is shown (*n* = 3).

##### Theoretical Background of SHG

The intensity of SHG light generated from a population of SHG molecules under irradiation by a fundamental beam, for a given polarization combination of the input and output beams, is given as:

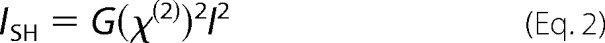
 where *I*_SH_ is the measured second-harmonic intensity, *G* is a function that depends on the experimental geometry, polarization of the fundamental and second-harmonic beams, refractive indices, angle of incidence, optical wavelengths, and the symmetry-allowed elements of the tensor nonlinear susceptibility χ^(2)^, and *I* is the intensity of the fundamental light. χ^(2)^ connects the microscopic properties of the second-harmonic active molecules to the observed second-harmonic intensity as,

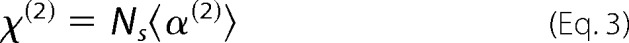
 where *N_s_* is the surface density of the second-harmonic active molecules and the angle brackets denote an orientational average over α^(2)^, the molecular nonlinear polarizability, a property of the second-harmonic active molecule that determines the probability of producing one second-harmonic photon from two photons of the fundamental beam. In solution phase with isotropic molecular orientation, SHG vanishes, as seen from Equation 3, but at surfaces this symmetry is broken, producing a net orientation and allowing SHG. The requirements for generating second-harmonic light are thus as follows: the molecules must possess second-harmonic activity (a non-zero α^(2)^) and a net, average orientation. Non-centrosymmetric molecules with a large difference dipole moment between the ground and excited states often possess second-harmonic activity. Equation 3 can be expressed further as,

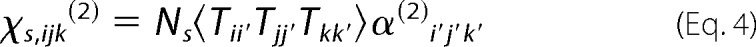
 where α^(2)^*_i_*_′_*_j_*_′_*_k_*_′_ is the molecular nonlinear polarizability measured in the cartesian coordinate frame of the molecule. It is common for planar second-harmonic active molecules, such as dye molecules with aromatic heterocycles, to exhibit nonlinear polarizability in a single plane, such as *x*′*z*′, and mirror symmetry perpendicular to the *x*′ axis of the molecule. If molecular orientation is also isotropic in the plane of the surface, as is commonly the case, by symmetry only three terms of χ*_s,ijk_*^(2)^ contribute to the observed SHG, χ*_ppp_*^(2)^ χ*_pss_*^(2)^ χ*_sps_*^(2)^. Furthermore, the nonlinear polarizability is often dominated by a single element, *i.e.* α′*_z_*_′_*_z_*_′_*_z_*_′_^(2)^. Then χ^(2)^ is given by,

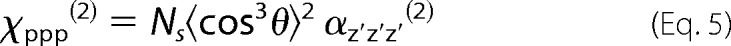
 and

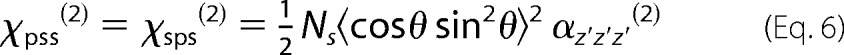
 where *p* and *s* denote the polarizations parallel and perpendicular to the plane of incidence and θ is the angle between the molecular *z*′ axis and the surface normal (48). The observed SHG intensity of the labeled protein from Equation 2 is thus directly related to the orientationally averaged tilt angle θ of the second-harmonic active moiety in the laboratory frame. For example, SHG is often measured using total internal reflection geometry. Under total internal reflection excitation at the critical angle, under *p-*polarized excitation and detection, and with a population of oriented molecules that have a single dominant element in nonlinear polarizability α*_z_*_′_*_z_*_′_*_z_*_′_ and a narrow orientational distribution, the SHG intensity *I*_SH_ ≈ cos^6^θ. With this sensitivity to tilt angle, at a mean tilt angle of 45°, for example, and a population of oriented molecules, conformational changes of 1° or less could be resolved with typical experimental noise of ∼1%. For a dye of 1-nm length, this corresponds to a conformational arc of sub-Ångstrom length, which gives the technique great sensitivity.

## Results

### 

#### 

##### Ligand-induced α-Synuclein Conformational Change Analysis

**FIGURE 2.**
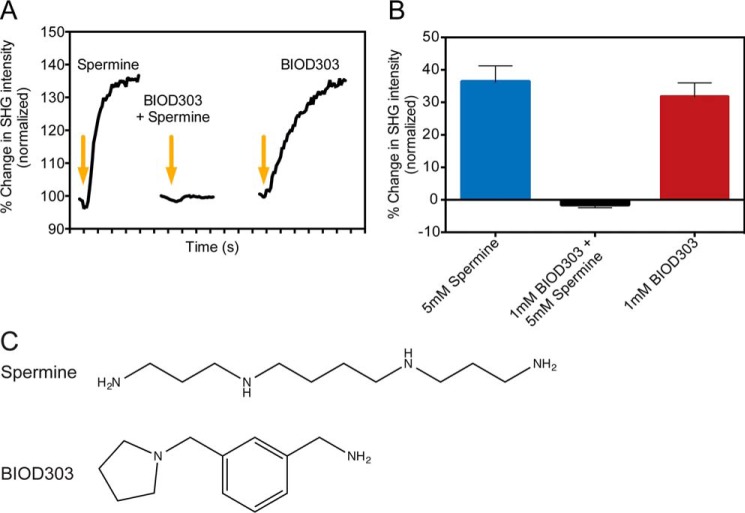
**SHG results of the α-synuclein assay and Maybridge Ro3 fragment library screen.** *A, left*, the SHG response for α-synuclein upon addition of 5 mm spermine. *Middle*, the SHG response of α-synuclein upon addition of 5 mm spermine after preincubation with 1 mm BIOD303. *Right*, the SHG response of α-synuclein upon addition of 1 mm BIOD303. The *arrows* denote compound addition. Each interval on the time axis is 10 s. *B,* quantification of the mean percent change in SHG intensity for 5 mm spermine, 5 mm spermine addition after preincubation with 1 mm BIOD303, and 1 mm BIOD303. The change in SHG intensity is plotted as the percent change normalized to preinjection levels. *C,* the chemical structures of spermine (*top*) and the compound BIOD303 (*bottom*). For all figures *error bars* = mean ± S.E., *n* = 3.

We began by examining the conformational change of α-synuclein upon addition of the tool compound spermine, which has previously been shown to bind to the C-terminal domain of α-synuclein and induce a change in the conformation of the protein from a closed to an open form (19, 40). Addition of 5 mm spermine to labeled α-synuclein resulted in an instantaneous increase in the SHG signal intensity (Fig. 2*A*, *left trace*). We measured the change in the SHG intensity at 5 mm spermine to be 36.3 ± 5.0% (Fig. 2*B*). We also tested two other known binders, spermidine and PcTS (25, 40, 49), and confirmed they also induced conformational change upon binding α-synuclein (3 mm spermidine = 19.9 ± 2.4%; 10 μm PcTS = −79.5 ± 6.2%, data not shown). Next, we tested each compound from the Maybridge Ro3 fragment library for an ability to inhibit the α-synuclein conformational change upon spermine addition. A hit was defined as complete abolishment of the spermine-induced signal change following preincubation of the test compound. One compound from the library, BIOD303, completely prevented spermine-induced conformational change at a concentration of 1 mm as measured by SHG (−1.4 ± 1.1%; Fig. 2*A*, *middle trace*; Fig. 2*B*).

We next sought to determine by SHG if BIOD303 could induce a conformational change in α-synuclein on its own. As shown in Fig. 2*A* (*right trace*), addition of 1 mm BIOD303 to α-synuclein changed the SHG baseline signal immediately. We measured the change in SHG intensity to be 31.7 ± 4.3% and classified the response of the compound by direction of signal relative to baseline, magnitude, and kinetics, to be similar to that of spermine (Fig. 2*B*). Although different conformational changes generally produce different changes in SHG magnitude, direction, or kinetics, they can lead to a similar change, as observed when BIOD303 and spermine bind to the protein. Only a single site (A90C) is labeled, so the angular change at this particular site may be similar although the overall conformations are distinct. Moreover, spermine and BIOD303 are not chemically similar (Fig. 2*C*).

**FIGURE 3.**
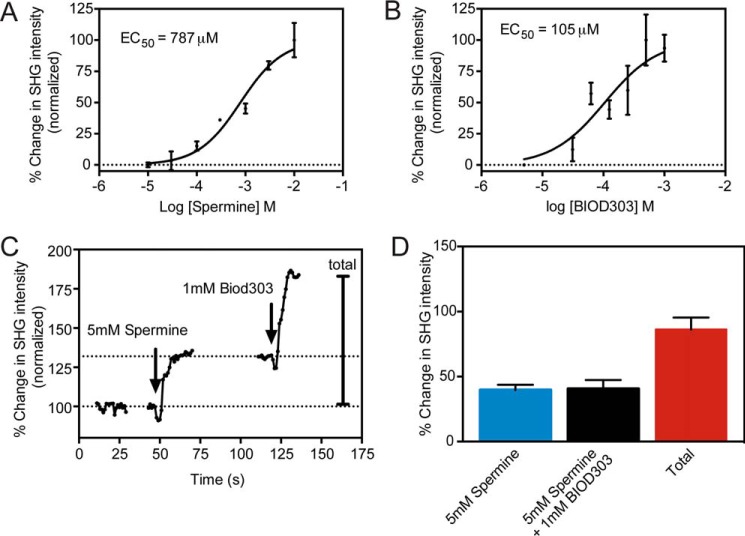
**BIOD303 induces a distinct conformation in α-synuclein compared with spermine.** *A,* dose-response curves of spermine; and *B,* BIOD303 on α-synuclein conformation as measured by SHG. *C,* a representative trace showing the SHG response from a competition experiment in which 5 mm spermine is injected onto α-synuclein prior to BIOD303 addition. *Left*, the SHG response for α-synuclein upon addition of buffer. *Middle*, the SHG response upon addition of 5 mm spermine onto α-synuclein. *Right*, the SHG response of α-synuclein upon addition of 1 mm BIOD303 after injecting 5 mm spermine. The *arrows* denote compound addition. The *dotted lines* on the graph represent the baseline level for normalization of the percent change in SHG intensity. Only the portion of the kinetic trace up to the SHG intensity plateau is shown. *D,* quantification of the mean percent change in SHG intensity for 5 mm spermine, 1 mm BIOD303 addition after preincubation with 5 mm spermine, and the total percent change for the original SHG baseline signal after both injections. Change in SHG intensity plotted as percent change normalized to preinjection levels. For all figures *error bars* = S.E. *n* = 5.

To better understand the relationship between spermine and BIOD303, we determined the EC_50_ of both compounds for α-synuclein conformational modulation using SHG. We obtained a dose-response curve (Fig. 3*A*) for spermine and determined its EC_50_ to be 787 μm (log EC_50_ −3.13), in agreement with previous values (40). We also measured the dose response of BIOD303 by SHG (Fig. 3*B*) and determined its EC_50_ to be 105 μm (log EC_50_ −3.98). Thus, BIOD303 has a ∼7.5-fold higher affinity for α-synuclein than spermine, which suggests that BIOD303 can potentially outcompete spermine in binding to α-synuclein.

To determine whether BIOD303 was competing with spermine for binding to α-synuclein, we repeated our original competition experiments except that we reversed the order of compound addition. In this experiment, if both ligands at a saturating dose bind to and produce the same conformation of α-synuclein, addition of BIOD303 subsequent to spermine would not be expected to change the SHG signal. As seen in Fig. 3*C*, addition of 5 mm spermine to α-synuclein resulted in a 39.8 ± 4.1% increase in SHG signal of α-synuclein, similar to previous results. Interestingly, subsequent addition of 1 mm BIOD303 to the same sample resulted in a further 40.7 ± 6.7% increase in the SHG intensity of α-synuclein suggesting that BIOD303 causes a different conformational change upon binding α-synuclein than spermine. If the time scale for protein and dye re-orientation back to the original apo state is slower than BIOD303 binding, *i.e.* the reaction is not at equilibrium, this would account for the additional change observed when BIOD303 is introduced after spermine. Thus the data suggests the lack of response in the original competition experiment from the screen is due to direct inhibition of spermine by BIOD303 and not because the sensitivity of the assay has reached a maximal response.

**FIGURE 4.**
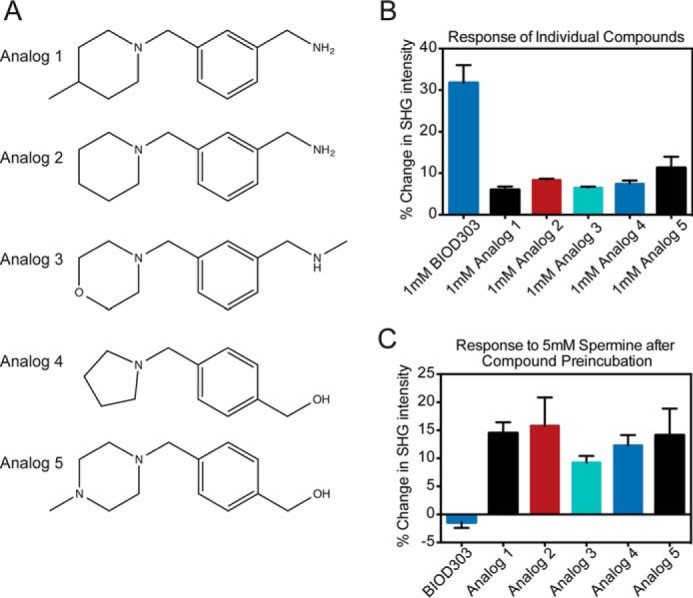
**BIOD303 is a highly specific inhibitor of the spermine-induced conformational change in α-synuclein.** *A,* chemical structures of the top five scoring BIOD303 analogs from the Maybridge Ro3 library as determined by the program SimFinder. *B,* quantification of the conformational change of α-synuclein upon exposure to the top five scoring chemical analogs of BIOD303. Each analog was added at 1 mm final concentration and the change in SHG intensity was measured. Representative data indicating the typical SHG response for 1 mm BIOD303 addition is included for comparison purposes. Changes in SHG intensity are plotted as the percent change normalized to pre-injection levels. *C,* quantification of conformational change in the analog-spermine competition assay. The indicated BIOD303 analog was preincubated with 1 mm α-synuclein and the change in SHG intensity upon exposure to 5 mm spermine was measured. Representative data indicating the typical SHG response from the BIOD303 + 5 mm spermine competition assay is included for comparison purposes. Changes in SHG intensity are plotted as a percent change. For all experiments *error bars* = S.E. *n* = 3.

Next, we wanted to determine whether fragments chemically similar to BIOD303 could also modulate the conformation of α-synuclein. We used the ranking program SimFinder (50) to select the five most closely related chemical analogs of BIOD303 from the Ro3 library (Fig. 4*A*). The addition of each individual analog at 1 mm resulted in a smaller increase in SHG intensity than BIOD303, indicating modulation of α-synuclein conformation (Fig. 4*B*, Table 1), but they did not fully block spermine binding in the competition assay (Fig. 4*C*, Table 1). These data support the exclusion of the analogs as hits in the original screen and suggest that the magnitude and direction of SHG responses can be used to distinguish structure-activity relationships, by measuring the degree to which ligands induce or prevent conformational change. In addition, these results demonstrate that BIOD303 is the most specific and potent inhibitor of spermine-induced conformational change from this series of compounds.

**TABLE 1:** **Quantification of BIOD303 analog data**

Compound	% Change	
	Compound only	Competition assay
BIOD303	31.7 ± 4.3%	−1.4 ± 1.1%
Analog 1	6.0 ± 0.8%	14.5 ± 1.9%
Analog 2	8.3 ± 0.4%	15.8 ± 5.1%
Analog 3	6.5 ± 0.3%	9.2 ± 1.7%
Analog 4	7.4 ± 0.8%	12.3 ± 1.9%
Analog 5	11.2 ± 2.7%	14.1 ± 4.8%

##### NMR Analysis of the Ligand-induced α-Synuclein Conformational Changes

**FIGURE 5.**
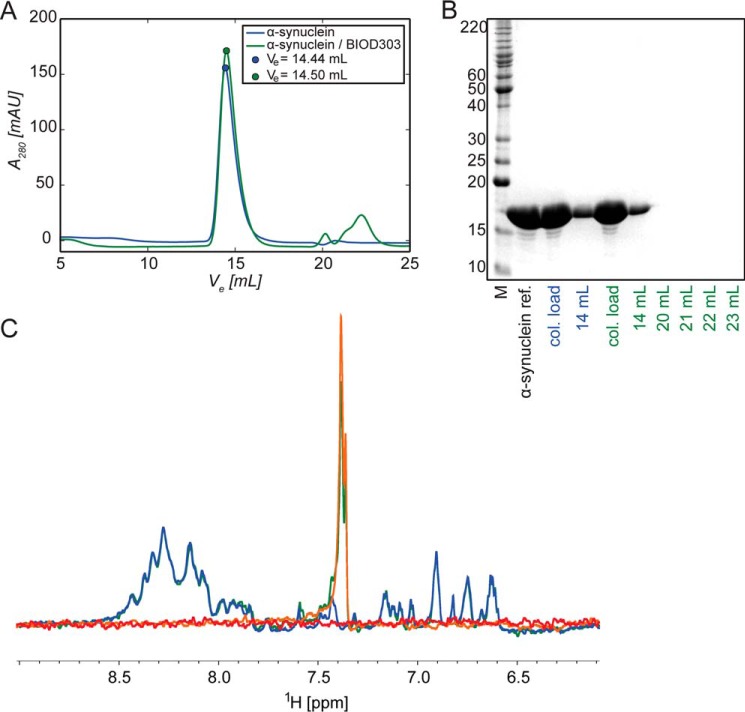
**Addition of a 5-fold excess of BIOD303 to α-synuclein does not alter its monomeric conformation in solution.** *A,* elution profile of α-synuclein (100 μm) in the absence (*blue*) and presence (*green*) of 5-fold excess of BIOD303 (500 μm) shows comparable retention behavior and elution at a volume of ∼14 ml on a Superdex 200 10/300 GL size exclusion column. *B,* different elution fractions of size exclusion chromatography and the column loads were analyzed by reducing SDS-PAGE and compared with an α-synuclein standard. *C,* ^1^H NMR spectra of α-synuclein (30 μm) in the absence (*blue*) and presence (*green*) of 5-fold excess of BIOD303 (150 μm) as well as BIOD303 (150 μm, *orange*) and HEPES buffer (*red*) as controls. Spectra were recorded at 700 MHz.

**FIGURE 6.**
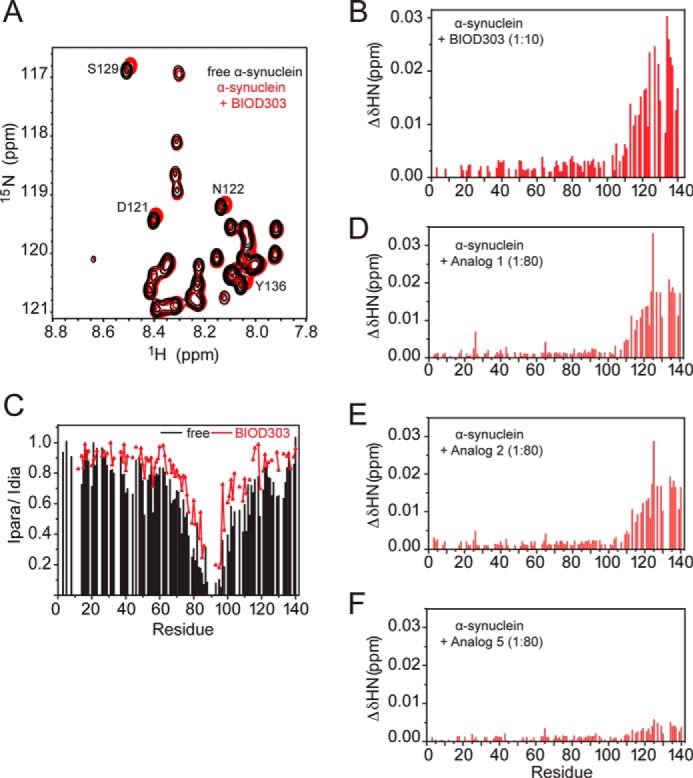
**BIOD303 binds to the C-terminal domain of α-synuclein and attenuates intramolecular long-range contacts.** *A,* detail of the superposition of ^1^H-^15^N NMR spectra of 100 μm α-synuclein in the absence (*black*) and presence (*red*) of BIOD303. *B*, chemical shift perturbation analysis of ^1^H-^15^N resonances of α-synuclein in the presence of BIOD303 at a 1:10 molar ratio. *C,* profile of PRE of amide protons in MTSL-labeled A90C α-synuclein (100 μm) in the absence (*black*) and presence (*red*) of BIOD303 (3 mm). *D–F*, chemical shift perturbation analysis of ^1^H-^15^N resonances of α-synuclein in the presence of analogs 1 (*D*), 2 (*E*), and 5 (*F*), at a molar ratio of 1:80.

To obtain further insight into the α-synuclein-BIOD303 interaction, we used NMR spectroscopy. NMR resonances are sensitive probes of protein-protein and protein-ligand interactions and can provide a description of interaction interfaces and binding affinities (51). First, the influence of BIOD303 on the aggregation state of α-synuclein was tested. To this end, we compared the elution profile of α-synuclein on a Superdex 200 10/300 GL in the absence and presence of a 5-fold excess of BIOD303 (Fig. 5, *A* and *B*). The presence of BIOD303 did not change the retention time, indicating that α-synuclein remains predominantly monomeric. Consistent with this hypothesis, the overall signal intensity in a one-dimensional NMR spectrum of α-synuclein was not attenuated upon addition of a 5-fold molar excess of BIOD303 (Fig. 5*C*). Next, BIOD303 was exposed to ^15^N-labeled α-synuclein and changes in chemical shifts were followed by two-dimensional ^1^H-^15^N correlation spectra of α-synuclein (Fig. 6*A*). Significant changes in NMR signals (∼0.01%) were observed for residues Gly-111 to Ala-140 of α-synuclein (Fig. 6, *A* and *B*) at a 10:1 compound:protein molar ratio. A similar perturbation profile had been observed upon addition of spermine to α-synuclein (40), indicating that both BIOD303 and spermine bind to the C-terminal domain of α-synuclein.

We also tested analogs 1, 2, and 5 for binding to α-synuclein. The analysis of chemical shift differences suggest that analogs 1 and 2 bind α-synuclein more weakly than BIOD303 at 12-fold molar excess to α-synuclein (data not shown). No chemical shift was detected for analog 5 at 12-fold molar excess to α-synuclein, although chemical shift perturbation at the C terminus was observed at 80-fold molar ratio. The residual binding of analogs 1 and 2 at the α-synuclein C terminus was also emphasized at 80-fold molar ratio of compound:protein (Fig. 6, *D–F*). These data further corroborate our initial screening results and suggest that SHG is highly sensitive to even weak protein-ligand interactions.

Next, we studied the consequences of BIOD303 binding on the conformational ensemble populated by α-synuclein in solution using PRE. The interaction between a specifically attached paramagnetic nitroxide radical and nearby protons (less than ≈ 25 Å) causes broadening of their NMR signals because of an increase in transverse relaxation rate (19). This effect has an *r*^−6^ dependence on the electron-proton distance and thus allows the detection of long-range interactions in proteins. To observe transient long-range contacts within α-synuclein in the presence of BIOD303, we attached a paramagnetic MTSL label at position 90 in α-synuclein. A comparison of the PRE profiles of MTSL-A90C α-synuclein in the absence and presence of BIOD303 revealed decreased paramagnetic broadening at residues 40–46 and the C-terminal region covering residues 124–136 (Fig. 6*C*). Thus, BIOD303 binds to a similar site as spermine or to one with substantial overlap with it, and induces a conformational change by attenuating the long-range interactions in α-synuclein.

##### Effect of BIOD303 on α-Synuclein Aggregation in Cells

**FIGURE 7.**
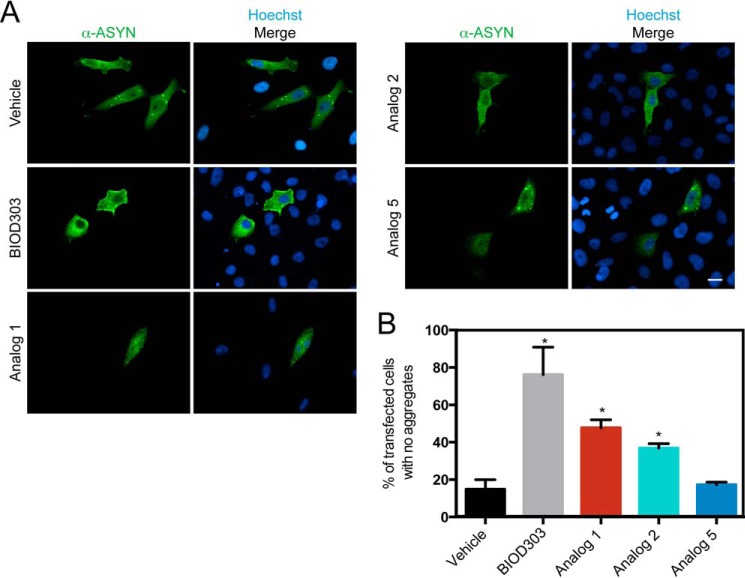
**BIOD303 inhibits α-synuclein inclusion formation in an H4 neuronal cell model.** *A,* representative images of transfected H4 cells treated with 500 μm of each compound. The staining for α-synuclein or the merge of α-synuclein and DNA (Hoechst) are indicated above the image. The compound addition is shown to the *left* of each image row. *Scale bar* = 20 μm. *B,* bar graph showing the percentage of H4 cells with no α-synuclein inclusions upon treatment with 500 μm of each compound. Student's *t* test (*, *p* < 0.05). *Error bars* = S.E. *n* = 2.

Because both SHG and PRE data indicated that BIOD303 altered the conformation of monomeric α-synuclein *in vitro*, we investigated the effect of BIOD303 on α-synuclein aggregation in cells. To do so, we tested BIOD303 in an established human H4 neuronal cell model of α-synuclein aggregation and inclusion formation (45, 46). In this model, a C terminally modified version of α-synuclein (SynT) is co-expressed with synphilin-1, resulting in the formation of LB-like, detergent-insoluble inclusions. The addition of 500 μm BIOD303 to H4 cells significantly increased the percentage of cells without α-synuclein inclusions (*, *p* = 0.0172, Student's *t* test) as shown by the absence of α-synuclein inclusions compared with control cells (Fig. 7). The effect on α-synuclein inclusion formation was dose dependent (data not shown). Analogs 1 and 2 also reduced inclusion formation (*, *p* = 0.0203, 0.0477 respectively, Student's *t* test), although to a lesser extent than BIOD303, consistent with the SHG and NMR data. Analog 5, which exhibited weak binding by NMR, had no effect on inclusion formation (*p* value = 0.7365, Student's *t* test). These results demonstrate that BIOD303 and analogs 1 and 2 directly target α-synuclein and reduce inclusion formation in a cellular context.

**FIGURE 8.**
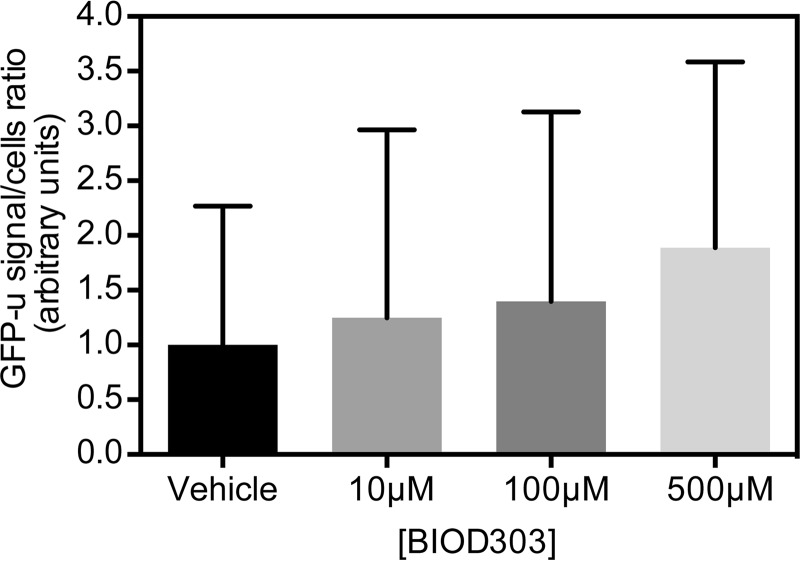
**BIOD303 does not significantly alter proteasome activity in H4 cells.** H4 cells expressing SynT + synphilin were transfected with a plasmid encoding GFP-u, a reporter of proteasome activity. Twenty-four hours after transfection, cells were treated with different concentrations of BIOD303. No significant differences could be detected between treated and untreated cells. *Error bars* = S.E. *n* = 2.

Because inclusion formation is a complex, multistep process we further investigated the mechanism by which BIOD303 reduced α-synuclein inclusion formation. We began by determining whether BIOD303 affected proteasome activity, which in turn could affect α-synuclein inclusion formation. To address this we used an unstable version of GFP (GFPu) that is targeted for proteasomal degradation (47). If proteasome activity is compromised, GFP-u accumulates in the cell, leading to an increase in fluorescence signal. In our experiments, we found no significant difference in the GFP signal of control cells compared with cells treated with different concentrations of BIOD303, suggesting that proteasome activity was not significantly affected (Fig. 8).

**FIGURE 9.**
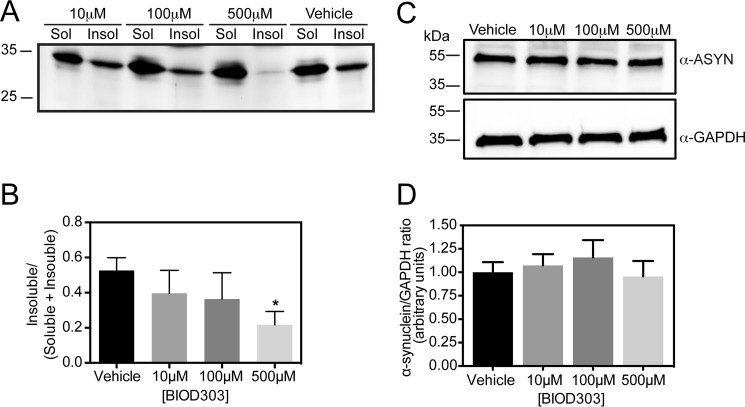
**BIOD303 reduces the level of insoluble α-synuclein in H4 cells.** *A,* a representative Western blot of the Triton X-100 solubility assay comparing the soluble and insoluble fractions of α-synuclein upon BIOD303 treatment. Cells treated with 500 μm BIOD303 show a significant reduction in the amount of α-synuclein in the insoluble (*Insol*) fraction. *B,* bar graph quantifying the ratio of insoluble α-synuclein to total α-synuclein from the Triton X-100 solubility assay. H4 cells treated with different concentrations of BIOD303 show a dose-dependent reduction in the amount of α-synuclein in the insoluble (*Insol*) fraction. Student's *t* test; *, *p* < 0.05. *n* = 3. *C,* representative immunoblot; and *D,* quantification showing that expression levels of α-synuclein do not change upon treatment with BIOD303. *Error bars* = S.E. *n* = 3.

To assess whether the observed effect on inclusion formation was indeed due to a change in the biochemical state of α-synuclein, we performed Triton X-100 solubility assays. We found that BIOD303 reduced the amount of detergent-insoluble α-synuclein compared with control cells (*, *p* = 0.0424, Student's *t* test) (Fig. 9, *A* and *B*), without changing the total levels of α-synuclein expression (Fig. 9, *C* and *D*), as assessed in a model where SynT and synphilin-1 are expressed in a 1:1 ratio (hence the slight difference in the apparent size on the SDS-PAGE when compared with the detergent solubility assay). This result, in combination with the *in vitro* biochemical and SHG experiments, indicates that BIOD303 reduces α-synuclein inclusion formation in cells, by binding to and directly modulating the conformation of monomeric α-synuclein.

## Discussion

Here we present an SHG-based approach to study and identify protein-small molecule conformational changes. Our goal was to test the hypothesis that modulating the monomeric α-synuclein conformation can affect aggregation of the protein in cells. SHG has previously been used to study the natural polarizability of biological materials such as collagen. In the pioneering studies by Freund *et al*. (52), SHG was used to demonstrate the highly ordered, anisotropic organization of collagen fibers in rat tail tendon. The intrinsic SHG activity of biological materials has also been demonstrated in other biological samples including tetra fish keratocytes and the nematode *Caenorhabditis elegans* (53, 54). Other applications of SHG include study of the cell membrane potential through the exogenous incorporation of organic second-harmonic active membrane dyes (55). The technique presented here renders a protein second-harmonic active by coupling a second-harmonic active dye to it via standard chemistries. Conjugation with an second-harmonic active probe offers the key advantage that the target protein can be modified either randomly at lysine residues using amine labeling or site specifically at either naturally occurring or engineered cysteine residues via maleimide labeling. In addition, attaching the protein directly to a surface ensures that the requirement for noncentrosymmetric orientation of the dye, necessary for SHG, is met. Taken together, the approach described here should allow the study of virtually any biological target via SHG.

To probe the role of conformation in α-synuclein aggregation and PD pathogenesis, we used SHG to identify a single compound, BIOD303, as a potent conformational modulator of the protein *in vitro*. The fragment appears to bind to a conformation of monomeric α-synuclein distinct from that bound by spermine, and it significantly reduces α-synuclein aggregation in a neuronal cell model. BIOD303 is distinct from other previously identified α-synuclein aggregation inhibitors such as curcurmin, epigallocatechin gallate, and hydroxyquinolines, which are known pan-assay interference compounds (26, 29, 56–58) in that it specifically binds to monomeric α-synuclein. In addition, BIOD303 has drug-like properties and appears to be an excellent starting point for medicinal chemistry efforts. The ability of SHG to identify BIOD303 and distinguish it from closely related analogs that bind to an IDP target that is challenging to screen against, for which few specific ligands are known, illustrates the sensitivity of the technique. As the magnitude and sign of the measured SHG change depend on the specific conformation and therefore the tilt angle of the label produced by binding each ligand, the technique can also classify ligands by the different conformations to which they bind. Proteins exist in an ensemble of conformations, particularly in the case of IDPs such as α-synuclein. Many of these conformations are short-lived and represent only minor populations in the total ensemble, yet they may nonetheless be crucially important to the function of a protein and also unique across a protein family. Therefore, a key potential advantage of conformational modulators as therapeutics is that they can target specific, conserved conformations and thereby act more selectively (1, 59–61). In summary, our results indicate that the conformation of monomeric α-synuclein plays an important, possibly decisive, role in the aggregation of the protein, and presumably PD pathogenesis. As shown here, small molecules that modulate α-synuclein conformation can be identified rapidly by SHG. By modulating the conformation of the monomeric protein, selective therapeutics directed to suppressing protein aggregation and PD pathogenesis at the earliest stage could be developed.

## Author Contributions

B. M. performed the SHG experiments, data analysis, and wrote the paper. G. Y. performed NMR data acquisition and NMR analysis. D. F. L. performed cell culture experiments, analysis, and wrote the paper. T. S. performed the SEC and NMR experiments. F. M. performed NMR data acquisition and analysis. K. G. contributed sample preparations. S. B. supervised sample production and wrote the paper. T. F. O. supervised the cell culture work and wrote the paper. M. Z. supervised the NMR work and wrote the paper. J. S. designed and supervised the project, performed the screen, and wrote the paper.

## Footnotes

4 The abbreviations used are:

IDP: intrinsically disordered protein
SHG: second-harmonic generation
PcTS: phthalocyanine tetrasulfonate
Ro3: rule of three
PD: Parkinson disease
PRE: paramagnetic relaxation enhancement
MTSL: oxy-2,2,5,5-tetramethyl-d-pyrroline-3-methyl)-methanethiosulfonate
DMSO: dimethyl sulfoxide.
